# Association between self-perception of aging, view of cancer and health of older patients in oncology: a one-year longitudinal study

**DOI:** 10.1186/s12885-017-3607-8

**Published:** 2017-09-02

**Authors:** Sarah Schroyen, Pierre Missotten, Guy Jerusalem, M. Van den Akker, F. Buntinx, Stéphane Adam

**Affiliations:** 1 Psychology of Aging Unit, Department of Psychology, University of Liège (ULg), Traverse des Architectes (B63c), 4000 Liege, BE Belgium; 2 INSERM U12919 Bordeaux Population Health, University of Bordeaux, Bordeaux, France; 3 Laboratory of Medical Oncology, University of Liège, Liège, Belgium; 4 Department of Medical Oncology, CHU Sart-Tilman Liège, Liège, Belgium; 5 Department of General Practice, KU Leuven, Leuven, Belgium; 6 CAPHRI Research School, Maastricht University, Maastricht, The Netherlands

**Keywords:** Ageism, Cancer, Oncology, Elderly, Self-perception, Stigmas

## Abstract

**Background:**

Identifying older people affected by cancer who are more at risk of negative health outcomes is a major issue in health initiatives focusing on medical effectiveness. In this regard, psychological risk factors such as patients’ perception of their own aging and cancer could be used as indicators to improve customization of cancer care. We hypothesize that more negative self-perception of aging (SPA) and view of cancer could be linked to worse physical and mental health outcomes in cancer patients.

**Methods:**

One hundred one patients diagnosed with cancer (breast, gynecological, lung or hematological) were followed for 1 year. They were evaluated on four occasions (baseline, 3, 6 and 12 months after the baseline). Their SPA, view of cancer and health (physical and mental) were assessed at each time of evaluation.

**Results:**

Negative SPA and/or view of cancer at baseline are associated with negative evolution of patients’ physical and mental health. Moreover, when the evolution of SPA and cancer view were taken into account, these two stigmas are still linked with the evolution of mental health. In comparison, only a negative evolution of SPA was linked to worse physical health outcomes.

**Conclusions:**

Such results indicate that SPA and view of cancer could be used as markers of vulnerability in older people with cancer.

**Electronic supplementary material:**

The online version of this article (10.1186/s12885-017-3607-8) contains supplementary material, which is available to authorized users.

## Background

Cancer is a very common disease: in Europe, 3.45 million new cases were diagnosed in 2012 [1]. Among patients suffering from cancer, older individuals represent a substantial proportion. It is estimated that in 2030, 70% of diagnosed cancers in the United States will affect patients older than 65 years [2]. Consequently, anticipating older cancer patients with higher risks of more negative outcomes is a major issue in efforts aimed at medical effectiveness and support of clinicians’ decision-making. In this perspective, it is now accepted that a Comprehensive Geriatric Assessment or Multidisciplinary Geriatric Assessment is useful to guide treatment decisions for older people with cancer. Such measures characterize the “functional age” of patients. Functional age differs from chronological age and accounts for physiological, social, and cognitive age-related changes which vary between individuals [3]. These geriatric assessments classically contain the essential domains evaluated by geriatricians, including functional status, comorbidities, cognition level, nutritional status, psychological status, and social support [4]. Each of these domains are independent predictors of morbidity and mortality in older patients [5–9].

These objective and medical parameters could, however, be advantageously complemented by an assessment of psychological risks all along the course of the disease. In this regard, self-perception of aging (SPA) could be a useful parameter to take into account. Indeed, several longitudinal studies on non-pathological aging have shown that the SPA of older patients could be an important predictor of their health evolution and even their longevity [10]. For example, in a longitudinal study involving 385 individuals over 38 years, it was observed that participants with more negative age stereotypes had a 30.2% greater decline in memory performance than participants with less negative age stereotypes [11]. In another study lasting 18 years, it was demonstrated that participants with a more negative SPA reported worse functional health during follow-ups in comparison with those with a more positive SPA [12]. Similar results were observed when objective cardiovascular events were used as parameters (angina attacks, strokes, etc.). Indeed, in a 38–year study, 25% of participants in the negative age stereotypes group experienced a cardiovascular event, in comparison to 13% in the positive age stereotypes group [13]. Moreover, another 23-year study has shown that SPA is associated with longevity, as older individuals with an initial more negative SPA lived 7.5 years shorter than those with a more positive SPA [14]. Such relationships between ageism and accelerated decline of physical and mental health can notably be explained by the fact that people with a negative SPA were less likely to engage in healthy behaviors (e.g., having a healthy diet, engaging in physical exercise, minimizing alcohol or tobacco consumption, etc.) [15]. In addition, individuals that had been exposed to negative aging stereotypes had a weaker will to live [16, 17].

In regard to the negative influence of SPA in non-pathological contexts, we can reasonably ask ourselves whether these associations also apply in clinical populations such as in oncology patients. Besides, suffering from cancer can lead older people to experience more discriminative behaviors linked to their disease [18, 19]. As for ageist stereotypes, those linked to cancer are not without consequences for the patient. Indeed, patients having suffered from cancer stigmas were more likely to be depressed and to report a lower quality of life than those who felt less stigmatized [20, 21]. However, these studies on cancer stigma were: (1) cross-sectional, and therefore did not analyze the effects of such stigmas on the evolution of the disease, and (2) did not specifically focus on the older population.

In a previous study [22], we investigate the combined influence of these two kinds of stigmas - ageism and cancer stigmas - on 101 aged patients suffering from cancer. It was shown that negative SPA and/or view of cancer are linked to more negative global health conditions. More precisely, SPA was associated with physical and mental health whereas view of cancer was only related with mental health. Nevertheless, since these conclusions originated from cross-sectional data, the question of the effect of these stigmas on the evolution of health still remains unanswered. In the current study, we followed the same patients over 1 year. Based on mentioned studies in normal and clinical older population, we hypothesized that a double stigmatization - involving a negative SPA and view of cancer at the baseline and over time - was linked to a worse evolution of physical and mental health. In other words, we believe that this double stigmatization could be a marker of vulnerability in oncology, as negative SPA in normal aging.

## Methods

### Participants

One hundred one patients (*M* age = 73.5; *SD* = 6.2) participated in the study thanks to a collaboration between the department of medical oncology of the CHU Sart-Tilman Liège University Hospital (Belgium) and the Psychology of aging unit of the University of Liège. Written informed consent was obtained from the patients. Eligible patients were those over 65 years old with a sufficient knowledge of French, diagnosed with cancer (breast, lung, gynecological or hematological cancer) but without comorbid diagnosis of dementia, and had a treatment planned (i.e. surgery, chemotherapy, radiotherapy or hormonotherapy). We included all stages of cancer, as well as patients with a newly diagnosed cancer or relapse (these parameters were controlled in the analyses). For more details on the recruitment and characteristics of patients, see the cross-sectional study [22]. These patients were seen four times: at the baseline (T0, *n* = 101), after 3 months (T3, *n* = 75), 6 months (T6, *n* = 64) and 12 months (T12, *n* = 58). The decline in the number of patients after the one-year follow-up is explained by refusals (*n* = 22; 16 at T3, 5 at T6, 1 at T12), impossibility to reach the patient (*n* = 4; 2 at T3, 1 at T6 and 1 at T12) or death (*n* = 17; 8 at T3, 5 at T6 and 4 at T12). At baseline, all patients were met in the hospital (or day hospital). For the follow-ups, when possible, patients were also seen at the (day) hospital. Non-hospitalized patients or those who did not have an appointment scheduled at the time of the follow-up were seen at their home or interviewed by phone.

### Materials

- *Demographics and medical information.* Data were collected on age, sex, educational level and civil status at baseline. Medical information, such as the site, kind (initial or recurrent), stage of cancer and number of comorbidities were obtained through medical records at baseline. Additional information (treatment or death) was obtained during follow-ups.
- *Cognitive level* was assessed only at the baseline with the French version of the *Mini Mental State Examination* (MMSE) [23]. This test measures orientation, learning, attention, memory, language and constructive praxis.
- *SPA* was measured with the *Attitudes to Aging Questionnaire* (AAQ) [24], translated and validated in French [25]. Measurements were taken at each testing time point (T0, T3, T6 and T12). This scale was specifically developed to flexibly and comprehensively assess attitudes toward the aging process as a personal experience from the perspective of older adults. For each of the 24 items of the scale (α = .78), participants respond on a five-point Likert-type scale ranging from 1 (*strongly disagree/not at all true*) to 5 (*strongly agree/extremely true*). This scale can be divided into three subscales: Psychosocial loss, Physical change and Psychological growth. In the present study, we only used the total score (range: 24–120). A higher total score reflects more positive SPA.
- *View of cancer* was assessed using the *Social Impact Scale* (SIS) [26] translated into French. This scale was also administered at each testing time point. It measures the individual’s perception of being stigmatized because of cancer. Some items were slightly modified in order to adapt them to older people. More specifically, two items related to the work place (“My employer/co-workers have discriminated against me” and “My job security has been affected by my illness”) were rephrased in order to refer to “useful activities (voluntary work, baby-sitting…)” rather than paid activities. This scale comprises 24 items (α = .87) that are answered using a 4-point Likert-type scale ranging from 1 (*strongly disagree*) to 4 (*strongly agree*). Items can be divided into four subscales: Social rejection, Financial Insecurity, Internalized shame and Social isolation. As for the AAQ, we only used the total score (range: 24–96). Originally, a high score indicates a strong feeling of stigmatization. However, in order to simplify the reading of stigma results, the score was reversed: a higher score indicated a lower level of cancer stigma, similarly to the SPA (i.e. AAQ) scale. In other words, a higher SIS score meant a more positive view of cancer.
- *European Organization for Research and Treatment of Cancer Quality of Life Questionnaire Core 30 (EORTC QLQ-C30).* This 30-item instrument [27] was administered during the four time points. In agreement with Giesinger et al. [28], we excluded from data analyses one item measuring financial difficulties and two items measuring the quality of life. Based on the 27 remaining items, the questionnaire includes 5 functioning scales: (1) physical, (2) role, (3) emotional, (4) cognitive and (5) social. It also measures symptomatology with three scales (Nausea and Vomiting, Fatigue, Pain) as well as with 5 separate items (Dyspnoea, Insomnia, Appetite Loss, Constipation and Diarrhoea). All scores are transformed into a 0–100 scale. On this basis, a summary score of global health was calculated (α = .9). A higher score indicated better health. For conceptual matters, we also have distinguished physical and mental health as we have done for the cross-sectional study [22]. For physical health (α = .89), we have included the following parameters (19 items): (1) the physical and role functioning scales; (2) symptoms scales and single items. For mental health (α = .78), we have included the emotional, social and cognitive functioning scale (8 items).

A copy of the questionnaire used in this study can be found in supplemental files (see Additional file 1).

### Data analyses

First of all, characteristics of our sample were described for participants who completed the entire follow-up. Their SPA, view of cancer and health (global, mental and physical) were described at each time. Differences between baseline and T12 were tested with a paired *t*-test. We also compared baseline patients’ characteristics for those who completed all the follow-ups with those who were lost (refusal, death or unreachability) during the follow-up *(t*-test for continuous variables, or Chi-square test for categorical variables). Secondly, to examine our hypothesis that SPA and view of cancer at the baseline influence the evolution of health (global, physical and mental), we used longitudinal linear mixed models and estimated it with the R software (more precisely, random intercept slope model). In this model, each subject is assumed to have his/her own unique functional relation between the dependent variable and time-related predictor (i.e., random intercept). Therefore, subject-specific curves estimating the effect of treatments are fit to the data and the pooled (or average) effect is estimated. In other words, a curve that optimally fits the data for each given person is estimated [29]. This approach can handle missing data; thus, it allowed us to include participants who did not finish the follow-ups. Both the intercept and the slope were fitted as random effects, allowing them to vary between individuals. We considered the SPA score and view of cancer at the baseline and took into account the individual evolution (during a whole year) of participants’ health, by computing an estimate rate change of the health. As control variables, we included age, gender, educational and cognitive level, comorbidities, cancer’s main characteristics (kind, site and stage), occurrence of death (when applicable) and type of treatment (surgery, radiotherapy, chemotherapy, hormonotherapy or other kind of therapy). We did not include the health scores of participants at baseline as a fixed effect [30], nevertheless, we have used a random intercept, which control for participants’ different starting scores (or intercepts) on health scores. Using a backward-elimination strategy, we reduced covariates to those significant at .05, which presented the best-fit model. We finally verified whether this association was also present over time, by taking into account the evolution of SPA and view of cancer in relation to the evolution of health. In other words, we calculated the estimate range change of SPA, view of cancer and health during the year, while controlling for individual differences at the start of the study. For this purpose, we used linear mixed models that took into account the relation between each participant’s evolution of SPA and view of cancer (not only the baseline score, but the four subsequent measures: T0, T3, T6, T12), and the evolution of their health status, using the same covariates as in the previous analysis. We also reduced covariates to those significant at .05 with a backward-elimination strategy and a best-fit model. Raw data are avalaible in Additional file 2.

## Results

### Sample characteristics

Patients’ characteristics are presented in Table 1. The SPA, view of cancer and health (physical, mental and global) of participants at the one-year follow-up (for those who completed the study) were not significantly different from baseline (all *p* > .11). In comparison, patients who did not complete the study had a more negative SPA, although they were similar to the one-year follow-up participants in terms of view of cancer and global, physical and mental health. Furthermore, patients who died before the end of the study had a more negative SPA and view of cancer, as well as a worse global and physical health, compared to patients who completed the study. However, they did not differ on mental health.

**Table 1:** Descriptive characteristics of the sample

Characteristics		One-year follow-up group (*n* = 58)^a^ M (SD) or %	Lost group (*n* = 43) M (SD) or N %	Baseline comparison between the lost group and patients who completed the study *t* (*p*) or χ^2^ (*p*)
Women		54 (93.1%)	12 (27.9%)	8.18 (.004)
Age		71.77 (5.53)	75.06 (6.32)	−2.8 (.006)
Primary cancer site	Breast	31 (53.4%)	16 (37.2%)	7.55 (.056)
	Gynecology	16 (27.6%)	9 (21%)	
	Lung	8 (13.8%)	16 (37.2%)	
	Hematology	3 (5.2%)	2 (4.6%)	
Cancer staging	Non-metastatic	53 (91.4%)	28 (65.1%)	10.72 (.001)
	Metastatic	5 (8.6%)	15 (34.9%)	
Kind of cancer	Initial cancer	48 (82.8%)	40 (93%)	2.32 (.12)
	Recurrence or progressive	10 (17.2%)	3 (7%)	
Charlson Comorbidity Index		1.79 (1.33)	2.56 (2.14)	−2.2 (.03)
Cognitive functioning		27.84 (1.96)	26.83 (2.32)	2.35 (.02)
Global health	Baseline	79.68 (15.97)	73.8 (17.38)	1.75 (.08)
	After 3 months	75.26 (15.17)		
	After 6 months	76.66 (17.53)		
	After one year	80.36 (14.2)		
Mental health	Baseline	82.35 (17.7)	78.68 (19.72)	.956 (.34)
	After 3 months	79.66 (19.98)		
	After 6 months	82.17 (18.97)		
	After one year	83.24 (18.5)		
Physical health	Baseline	78.89 (17.46)	72.33 (18.43)	1.81 (.073)
	After 3 months	73.41 (16.9)		
	After 6 months	74.98 (18.28)		
	After one year	79.5 (15.04)		
SPA	Baseline	87.52 (12.79)	81.56 (13.36)	2.19 (.03)
	After 3 months	84.5 (12.64)		
	After 6 months	86.05 (13)		
	After one year	84.4 (14.26)		
Cancer view	Baseline	83.31 (9.55)	80.05 (11.17)	1.36 (.17)
	After 3 months	81.1 (10.89)		
	After 6 months	84.53 (10.38)		
	After one year	84.5 (12.04)		

^a^The entire sample at the baseline comprised 101 patients. For more information concerning the sample, see cross-sectional study [22]

### Mixed linear models

After backward elimination, we observed a significant effect of the baseline SPA score and view of cancer on the evolution of global health (see Table 2): a more negative SPA and/or view of cancer were associated with worse health outcomes (with all covariates included). In other words, patients’ SPA and/or view of cancer shortly after diagnosis seemed to be significantly associated with the evolution of their health the following year. Moreover, we noted that having a chemotherapy treatment, a metastatic cancer and being a female were linked with a worse evolution of global health. Concerning physical health (after backward elimination), a negative SPA and view of cancer at baseline were associated with a worse evolution of physical parameters (all covariates included). Again, having a chemotherapy treatment and being a woman were linked with a worse evolution of physical health. Moreover, negative physical outcomes were associated with hematological cancer (in comparison to breast cancer), initial cancers and greater number of comorbidities. For mental health, we also observed a significant link between negative SPA and view of cancer at baseline on negative mental health evolution. Issues of mental health were also increased in relation to chemotherapy treatment and gynecological cancer (in comparison to breast cancer).

**Table 2:** Best fit model for the estimate rate change of health after backward elimination for SPA and view of cancer, baseline scores

Characteristics		Global health			Physical health				Mental health				
		Coeff.	*SE*	*t*	*p*	Coeff.	*SE*	*t*	*p*	Coeff.	*SE*	*t*	*p*
SPA (baseline)		.38	.099	3.808	< .001	.37	.1	3.587	< .001	.53	.11	4.887	< .001
View of cancer (baseline)		.359	.13	2.735	< .001	.3	.14	2.204	.03	.49	.14	3.463	< .001
Carcinoma staging^a^		−7.37	3.34	−2.21	.03	-	-	-	-	-	-	-	-
Chemotherapy^b^		−7.31	1.57	−4.66	< .001	−7.49	1.74	−4.33	< .0001	−7.6	2.04	−3.718	< .001
Gender^c^		8.13	3.74	2.17	.03	13.08	5.05	2.591	.01	-	-	-	-
Cancer site^d^	Lung (vs breast)	-	-	-	-	−6.63	4.35	−1.525	.13	5	3.45	1.451	.15
	Gynecological (vs breast)	-	-	-	-	−4.54	3.16	−1.439	.15	−10.09	3.33	−3.03	.003
	Hematological (vs breast)	-	-	-	-	−17.68	6.3	−2.806	.006	.41	6.3	0.06	.95
Kind of cancer^e^		-	-	-	-	7.7	3.81	2.023	.046	-	-	-	-
Comorbidities		-	-	-	-	−1.69	.082	−4.33	< .001	-	-	-	-
REML criterion at convergence		2349.1				2375				2472			

^a^0 = non-metastatic, 1 = metastatic; ^b^0 = no treatment, 1 = treatment; ^c^0 = women, 1 = men; ^d^0 = breast cancer, 1 = lung, gynecological or hematological; ^e^0 = initial cancer, 1 = recurrent cancer

As depicted in Table 3, when we took into account the evolution of SPA and view of cancer over time in relation to the evolution of global health, these two stigma parameters were still associated with worse health outcomes (as for metastatic cancer and chemotherapy treatment). Nonetheless, the view of cancer was no longer significantly linked with physical health: a worse evolution is only associated, in the model, by a negative SPA, metastatic cancer and chemotherapy treatments. Finally, for mental health, both negative SPA and view of cancer were significant linked with more negative evolutions. More mental issues were also reported for metastatic cancers, chemotherapy treatments breast cancer (in comparison to lung cancer) and gynecological cancer (in comparison to breast cancer).

**Table 3:** Best fit model for the estimate rate change of health after backward elimination for the estimate rate change of SPA and view of cancer

Characteristics		Global health				Physical health				Mental health			
		Coeff.	*SE*	*t*	*p*	Coeff.	*SE*	*t*	*p*	Coeff.	*SE*	*T*	*p*
SPA		.41	.07	5.92	< .001	.4	.07	5.46	< .001	.53	.07	6.785	< .001
Cancer view		.17	.07	2.318	.02	-	-	-	-	.53	.09	5.98	< .001
Carcinoma staging^a^		−6.86	3.08	−2.23	.03	−7.78	3.44	−2.263	.02	−8.89	3.68	−2.41	.02
Chemotherapy^b^		−6.4	1.54	−4.142	< .001	−6.89	1.7	−4.05	< .0001	−5.73	1.91	−3.005	.003
Cancer site^c^	Lung (vs breast)	-	-	-	-	-	-	-	-	8.31	3.66	2.274	.03
	Gynecological (vs breast)	-	-	-	-	-	-	-	-	−7.39	3.03	−2.44	.02
	Hematological (vs breast)	-	-	-	-	-	-	-	-	7	5.7	1.227	.22
REML criterion at convergence		2344.5				2403.1				2425.7			

^a^0 = non-metastatic, 1 = metastatic; ^b^0 = no treatment, 1 = treatment; ^c^0 = breast cancer, 1 = lung, gynecological or hematological

## Discussion

Both cancer and aging can lead to stigmatization. In addition, these stigmas have been linked to more issues in mental and physical health. Indeed, longitudinal studies in normal aging have demonstrated deleterious consequences of negative SPA on physical and mental health: participants with a more negative SPA report worse functional health, more cardiovascular and memory issues and their longevity is reduced [11, 12, 14, 31]. Moreover, cancer stigmas lead to more depression and a lower quality of life [20, 21]. However, the association between such stigmas (age and cancer) and health consequences has not yet been studied for the elderly suffering from cancer. Therefore, the aim of this longitudinal study was to analyze the link between SPA, view of cancer and health outcomes, which would also refine the results obtained in a previous cross-sectional study on this issue [22].

As demonstrated in the present study, SPA and/or the view of cancer measured at the baseline were linked with a negative evolution of global health. In other words, SPA and cancer stigma measured shortly after the diagnosis of cancer is associated with the occurrence of negative health outcomes for these patients. More specifically, results showed that a negative SPA and/or view of cancer at baseline were associated with a negative evolution of physical and mental health. These results are in accordance with previous studies on stigmas related to health conditions. Indeed, concerning view of cancer, our data are in line with previous cross-sectional studies carried out in patients aged from 18 to 88 years old [20, 21, 32, 33]. Moreover, our SPA results are in accordance with previous long-term studies among “normal” older people [11, 12, 14, 31]. However, we have to point out that in the latter study an initial negative SPA was predictive of a negative evolution of health over long time periods (18 to 38 years). In comparison, in our study, the follow-up was much shorter (1 year). However, the significant relationship between stigmas and health outcomes over this short time period shows that the negative effects of SPA are observable in the short-term, and not only in the long run.

Furthermore, we analyzed the association between the evolution of SPA and view of cancer over time and the evolution of health. Concerning physical health, the relation between negative SPA and physical difficulties was significant. These results had already been observed in a previous study with “normal” older people, in which SPA significantly predicted functional health status over a period of 18 years [12]. By contrast, the evolution of the view of cancer was not related to the evolution of physical health. Regarding the evolution of mental health, our results showed that its link with SPA and/or view of cancer remained stable during the one-year follow-up.

In line with our hypothesis, the results of the present study indicate that SPA and view of cancer could well be seen as markers of vulnerability in elderly people suffering from cancer. Indeed, they are apparently associated with several components of physical and mental health and emerge as good predictors of a negative evolution of aging. Therefore, SPA and view of cancer could be considered among the risk factors of vulnerability, and added to those that are traditionally taken into account (functional status, cognition, etc.). In addition, SPA can be viewed as a vulnerability factor that is more global than other health parameters such as cognitive status. Indeed, previous studies have suggested that cognitive impairment could be a powerful prognostic factor of health deterioration, including mortality for older patients with cancer [34–36]. However, on the basis of longitudinal and experimental studies, Levy demonstrated that cognitive impairment is predicted by participants’ perception of aging [10]. Therefore, we suggest that SPA could be considered as a more global marker of vulnerability than cognition. However, additional studies should be performed to confirm this hypothesis. Moreover, the importance of considering SPA as a marker of vulnerability is that, in contrast to more traditional parameters (sex, comorbidities…), SPA is partially malleable and can be improved. More precisely, some interventions could counteract or minimize the effect of SPA on older patients suffering from cancer (activation of positive stereotypes, self-affirmation…for more information see [37]).

If our results confirm that a negative SPA and/or view of cancer were related to a negative evolution of physical and mental health, the question of causality between SPA, view of cancer and health still remains. Based on our results, we cannot determine whether it is negative health perception that leads to negative SPA and view of cancer or, the other way around, if negative SPA and view of cancer give rise to negative health perception. Although, to our knowledge, this relationship has never been studied in oncology, it was shown in a study involving women with multiple sclerosis that the attitudes towards aging were influenced by functional limitations [38]. Also, longitudinal studies in non-pathological aging (described previously) showed that self-perception of aging may predict the evolution of physical and mental health of the elderly [11–14]. Therefore, making the hypothesis of a bi-directional link between SPA/view of cancer and health seems plausible. In order to confirm that the SPA/view of cancer could be the cause of a more negative evolution of health, it would be necessary to design an interventional study in which the effect of a minimization of stigmas on the evolution of health would be analyzed. For instance, to improve the SPA, it is possible to use subliminal activation (perception without awareness) of positive stereotypes about aging. This methodology was used in the context of “normal” aging and results showed a positive impact of subliminal activation on: (1) general view of aging, (2) self-perception of aging, and (3) physical functioning [39].

In order to confirm our follow-up results, additional studies with longer follow-up periods and larger samples of old patients in oncology are necessary. Moreover, our analyses are based on subjective self-reported physical and mental health. For future studies, it will be interesting to see the effects of SPA and view of cancer on objective health parameters (e.g., mortality, cancer recurrence, biological parameters). For example, we can hypothesize that SPA and view of cancer could be predictors of mortality. In our study, SPA and view of cancer were more negative at the baseline for patients who died during the first year. However, this should be interpreted with caution, since our number of patients (in particular deceased patients; *n* = 17) was too small for the use of survival statistics such as Kaplan-Meier’s survival analyses or Cox proportional hazards. Consequently, a larger population and a longer follow-up seems necessary to precisely assess the link between SPA, view of cancer and mortality. Nevertheless, we can note that other studies have already addressed this type of link. Indeed, in a non-pathological context, individuals with more negative SPA lived 7.5 years shorter than those with positive SPA [14]. Similarly, self-directed stereotypes related to chronic illnesses (e.g. cancer, arthritis, diabetes, etc.) during old age increase the risk of mortality in old people suffering from those diseases [40]. Furthermore, it could also be interesting to study factors such as compliance to treatment or medical advice (such as diet plans). Indeed, in “normal” aging, Levy has shown that the relationship between ageism and accelerated health decline is notably explained by the fact that people with a negative SPA were less likely to engage in healthy behaviors [15]. In this regard, it would be interesting to analyze if a negative evolution of patients’ health could be partially explained by a diminution of compliance towards treatments linked to a more negative SPA.

## Conclusion

Our results showed that a negative SPA and/or view of cancer shortly after a cancer diagnosis were associated with increased reported difficulties of physical and mental health during a one-year follow-up. Even if the causality needs to be verified, this association suggests that SPA and view of cancer could constitute vulnerability factors affecting the evolution of health in oncology patients and, as such, they should be taken into consideration in personalized clinical practice.

## Additional files

Additional file 1: Questionnaire. Original questionnaire given to patients for this study (French language). (PDF 1004 kb)

Additional file 2: Raw data. All data generated or analyzed during this study. (XLS 90 kb)

## Abbreviation

SPA: Self-perception of aging
