# Supplementary material for: Association between self-perception of aging, view of cancer and health of older patients in oncology: a one-year longitudinal study
Source: BMC Cancer. 2017 Sep 2;17:614. doi: 10.1186/s12885-017-3607-8 (PMC5581442; doi:10.1186/s12885-017-3607-8)
Supplement: Supplementary file 1 — Questionnaire. Original questionnaire given to patients for this study (French language). (PDF 1004 kb) [file 12885_2017_3607_MOESM1_ESM.pdf]

Questionnaires

Facteurs influençant le vécu des personnes en oncogériatrie

Anamnèse

Informations générales

Sexe : F / M

Date de naissance : __ __ / __ __ / 19 __ __

Nationalité : ……………………………………………………………………………………………

Habitat :

- A domicile
  - Seul
  - Avec un conjoint
  - Avec la famille
- Résidence de service
- Maison de repos
- Autre :………………………

Etat civil :

- Célibataire
- Marié(e) / cohabitant(e) légal(e)
- Divorcé(e)
- Séparé(e)
- Veuf/Veuve

Niveau d'étude le plus élevé :

- Primaire
- Secondaire inférieur
  - général
  - technique
  - professionnel
- Secondaire supérieur
  - général
  - technique
  - professionnel
- Gradua : diplôme en ___________________
- Universitaire : diplôme en ___________________

Sujet n° : Date évaluation: …. /…. /20 ….

<u>Questionnaire AAQ</u> (Laidlaw et al., 2007)

Traduction : Adam, Crespin, Masson, Missotten, 2012

Instructions

Ce questionnaire vous demande comment vous ressentez le fait de vieillir.

Veuillez répondre à toutes les questions. Si vous n'êtes pas certain de la réponse à donner à une question, veuillez choisir celle qui semble la plus appropriée. Ce sera souvent votre première réponse.

Gardez à l'esprit vos attentes, vos espoirs, vos passions et vos préoccupations. Nous vous demandons de réfléchir à votre vie en général.

Les questions suivantes demandent à quel point vous êtes d'accord ou non avec les énoncés. Si vous êtes d'accord avec ceux-ci, entourez la réponse « Tout à fait d'accord ». Si vous n'êtes pas du tout d'accord avec les énoncés, encerclez la réponse « Pas du tout d'accord ». Si vous voulez indiquer que votre réponse se trouve quelque part entre « Tout à fait d'accord » et « Pas du tout d'accord », entourez une proposition entre les deux.

	Pas du tout d'accord	Pas d'accord	Incertain	D'accord	Tout à fait d'accord
1. En vieillissant, les gens sont davantage aptes à faire face à la vie.	Pas du tout d'accord	Pas d'accord	Incertain	D'accord	Tout à fait d'accord
2. C'est un privilège de vivre vieux.	Pas du tout d'accord	Pas d'accord	Incertain	D'accord	Tout à fait d'accord
3. La vieillesse est une période de vie synonyme de solitude.	Pas du tout d'accord	Pas d'accord	Incertain	D'accord	Tout à fait d'accord
4. La sagesse vient avec l'âge.	Pas du tout d'accord	Pas d'accord	Incertain	D'accord	Tout à fait d'accord
5. Vieillir apporte beaucoup de choses agréables.	Pas du tout d'accord	Pas d'accord	Incertain	D'accord	Tout à fait d'accord
6. La vieillesse est une période de vie synonyme de dépression.	Pas du tout d'accord	Pas d'accord	Incertain	D'accord	Tout à fait d'accord
7. Il est important de faire de l'exercice physique à tout âge.	Pas du tout d'accord	Pas d'accord	Incertain	D'accord	Tout à fait d'accord

Les questions ci-dessous demandent à quel point pour vous, les énoncés suivants sont vrais ou pas. Si ceux-ci sont extrêmement vrais pour vous, entourez la réponse « Tout à fait vrai ». Si pour vous les énoncés ne sont pas vrais, encerclez la réponse « Pas du tout vrai». Si vous voulez indiquer que votre réponse se trouve quelque part entre « Tout à fait vrai » et « Pas du tout vrai », entourez une proposition entre les deux.

	Pas du tout vrai	Pas tellement vrai	Moyennement vrai	Plutôt vrai	Tout à fait vrai
8. Vieillir a été plus facile que ce que je pensais.	Pas du tout vrai	Pas tellement vrai	Moyennement vrai	Plutôt vrai	Tout à fait vrai
9. Je trouve qu'il est plus difficile de parler de mes sentiments en vieillissant.	Pas du tout vrai	Pas tellement vrai	Moyennement vrai	Plutôt vrai	Tout à fait vrai
10. Avec l'âge, je m'accepte mieux.	Pas du tout vrai	Pas tellement vrai	Moyennement vrai	Plutôt vrai	Tout à fait vrai
11. Je ne me sens pas vieux.	Pas du tout vrai	Pas tellement vrai	Moyennement vrai	Plutôt vrai	Tout à fait vrai
12. Je perçois la vieillesse principalement comme une période associée à la perte.	Pas du tout vrai	Pas tellement vrai	Moyennement vrai	Plutôt vrai	Tout à fait vrai
13. Mon identité ne se définit pas en fonction de mon âge.	Pas du tout vrai	Pas tellement vrai	Moyennement vrai	Plutôt vrai	Tout à fait vrai
14. J'ai plus d'énergie que ce que j'espérais pour mon âge.	Pas du tout vrai	Pas tellement vrai	Moyennement vrai	Plutôt vrai	Tout à fait vrai
15. Je perds mon indépendance physique en vieillissant.	Pas du tout vrai	Pas tellement vrai	Moyennement vrai	Plutôt vrai	Tout à fait vrai
16. Mes problèmes de santé physique ne m'empêchent pas de faire ce que je souhaite.	Pas du tout vrai	Pas tellement vrai	Moyennement vrai	Plutôt vrai	Tout à fait vrai
17. En vieillissant, je trouve qu'il est plus difficile de se faire de nouveaux amis.	Pas du tout vrai	Pas tellement vrai	Moyennement vrai	Plutôt vrai	Tout à fait vrai
18. C'est très important pour moi de transmettre les bénéfices de mes expériences aux plus jeunes.	Pas du tout vrai	Pas tellement vrai	Moyennement vrai	Plutôt vrai	Tout à fait vrai

	Pas du tout vrai	Pas tellement vrai	Moyennement vrai	Plutôt vrai	Tout à fait vrai
19. Je pense que ma vie a eu un impact.	Pas du tout vrai	Pas tellement vrai	Moyennement vrai	Plutôt vrai	Tout à fait vrai
20. Je ne me sens plus aussi impliqué dans la société, maintenant que je suis plus âgé.	Pas du tout vrai	Pas tellement vrai	Moyennement vrai	Plutôt vrai	Tout à fait vrai
21. J'aimerais donner un bon exemple aux plus jeunes.	Pas du tout vrai	Pas tellement vrai	Moyennement vrai	Plutôt vrai	Tout à fait vrai
22. Je me sens exclus de certaines choses à cause de mon âge.	Pas du tout vrai	Pas tellement vrai	Moyennement vrai	Plutôt vrai	Tout à fait vrai
23. Ma santé est meilleure que ce que j'espérais pour mon âge.	Pas du tout vrai	Pas tellement vrai	Moyennement vrai	Plutôt vrai	Tout à fait vrai
24. Grâce à l'exercice physique, je reste actif et en forme.	Pas du tout vrai	Pas tellement vrai	Moyennement vrai	Plutôt vrai	Tout à fait vrai

<u>Questionnaire SIS</u> (Fife & Wright, 2000)

Traduction : Schroyen, Missotten, Adam, 2013

Instructions

Ce questionnaire vous demande comment vous ressentez le fait d'avoir un cancer.

Veuillez répondre à toutes les questions. Si vous n'êtes pas certain de la réponse à donner à une question, veuillez choisir celle qui semble la plus appropriée. Ce sera souvent votre première réponse.

Gardez à l'esprit vos espoirs, vos préoccupations, et vos expériences.

Les questions suivantes demandent à quel point vous êtes d'accord ou non avec les énoncés. Si vous êtes d'accord avec ceux-ci, entourez la réponse « Tout à fait d'accord ». Si vous n'êtes pas du tout d'accord avec les énoncés, encerclez la réponse « Pas du tout d'accord ». Si vous voulez indiquer que votre réponse se trouve quelque part entre « Tout à fait d'accord » et « Pas du tout d'accord », entourez une proposition entre les deux.

	Tout à fait d'accord	D'accord	Pas d'accord	Pas du tout d'accord
1. Les personnes avec qui je suis engagé dans des activités utiles non (ou peu) rémunérées (bénévolat, association, garde d'enfants,…) m'ont discriminé	Tout à fait d'accord	D'accord	Pas d'accord	Pas du tout d'accord
2. Certaines personnes agissent comme si j'étais moins compétent que d'habitude	Tout à fait d'accord	D'accord	Pas d'accord	Pas du tout d'accord
3. Je sens que j'ai été traité avec moins de respect que d'habitude par les autres	Tout à fait d'accord	D'accord	Pas d'accord	Pas du tout d'accord
4. Je sens que les autres s'inquiètent de pouvoir « attraper » ma maladie à travers un contact comme une poignée de main ou en mangeant la nourriture que je prépare	Tout à fait d'accord	D'accord	Pas d'accord	Pas du tout d'accord
5. Je sens que les autres m'évitent à cause de ma maladie	Tout à fait d'accord	D'accord	Pas d'accord	Pas du tout d'accord

	Tout à fait d’accord	D’accord	Pas d’accord	Pas du tout d’accord
6. Je sens que certains amis m’ont rejeté à cause de ma maladie	Tout à fait d’accord	D’accord	Pas d’accord	Pas du tout d’accord
7. Certains membres de ma famille m’ont rejeté à cause de ma maladie	Tout à fait d’accord	D’accord	Pas d’accord	Pas du tout d’accord
8. Je me retrouve dans des situations embarrassantes à cause de ma maladie	Tout à fait d’accord	D’accord	Pas d’accord	Pas du tout d’accord
9. A cause de ma maladie, les autres semblent bizarres et tendus quand ils sont autour de moi	Tout à fait d’accord	D’accord	Pas d’accord	Pas du tout d’accord
10. J’ai expérimenté des épreuves financières qui ont affecté ma vision de moi-même	Tout à fait d’accord	D’accord	Pas d’accord	Pas du tout d’accord
11. La sécurité de mes activités utiles non (ou peu) rémunérées (bénévolat, association, garde d’enfants,…) a été affectée par ma maladie	Tout à fait d’accord	D’accord	Pas d’accord	Pas du tout d’accord
12. J’ai expérimenté des épreuves financières qui ont affecté ma relation avec les autres	Tout à fait d’accord	D’accord	Pas d’accord	Pas du tout d’accord
13. Je sens que les autres pensent que je suis à blâmer pour ma maladie	Tout à fait d’accord	D’accord	Pas d’accord	Pas du tout d’accord
14. Je ne sens pas que je peux être ouvert avec les autres à propos de ma maladie	Tout à fait d’accord	D’accord	Pas d’accord	Pas du tout d’accord
15. J’ai peur que quelqu’un parle aux autres de ma maladie sans ma permission	Tout à fait d’accord	D’accord	Pas d’accord	Pas du tout d’accord
16. Je sens que j’ai besoin de garder ma maladie secrète	Tout à fait d’accord	D’accord	Pas d’accord	Pas du tout d’accord
17. Je sens que je suis au moins partiellement à blâmer pour ma maladie	Tout à fait d’accord	D’accord	Pas d’accord	Pas du tout d’accord
18. Je me sens mis à part des autres qui sont biens	Tout à fait d’accord	D’accord	Pas d’accord	Pas du tout d’accord
19. J’ai un plus grand besoin que d’habitude de m’assurer que les autres tiennent à moi	Tout à fait d’accord	D’accord	Pas d’accord	Pas du tout d’accord

	Tout à fait d’accord	D’accord	Pas d’accord	Pas du tout d’accord
20. Je me sens plus souvent seul que d’habitude	Tout à fait d’accord	D’accord	Pas d’accord	Pas du tout d’accord
21. A cause de ma maladie, j’ai le sentiment d’être inégal dans ma relation avec les autres	Tout à fait d’accord	D’accord	Pas d’accord	Pas du tout d’accord
22. Je me sens moins compétent que je l’étais avant ma maladie	Tout à fait d’accord	D’accord	Pas d’accord	Pas du tout d’accord
23. A cause de ma maladie, je me sens parfois inutile	Tout à fait d’accord	D’accord	Pas d’accord	Pas du tout d’accord
24. Des changements dans mon apparence ont affecté mes relations sociales	Tout à fait d’accord	D’accord	Pas d’accord	Pas du tout d’accord

Sujet n° : Date d'évaluation : …./ …. / 20…..

Questionnaire EORTC QLQ-C30 (Aaronson et al, 1993)

Traduction : Groupe EORTC

Nous nous intéressons à vous et à votre santé. Répondez vous-même à toutes les questions en entourant le chiffre qui correspond le mieux à votre situation. Il n'y a pas de « bonne » ou de « mauvaise » réponse. Ces informations sont strictement confidentielles.

Au cours de la semaine passée	Pas du tout	Un peu	Assez	Beaucoup
1. Avez-vous des difficultés à faire certains efforts physiques pénibles comme porter un sac à provision chargé ou une valise ?	1	2	3	4
2. Avez-vous des difficultés à faire une LONGUE promenade ?	1	2	3	4
3. Avez-vous des difficultés à faire un PETIT tour dehors ?	1	2	3	4
4. Etes-vous obligé(e) de rester au lit ou dans un fauteuil la majeure partie de la journée ?	1	2	3	4
5. Avez-vous besoin d'aide pour manger, vous habiller, faire votre toilette ou aller aux W.C. ?	1	2	3	4
6. Etes-vous limitée d'une manière ou d'une autre pour accomplir, soit votre travail, soit vos tâches habituelles chez vous ?	1	2	3	4
7. Etes-vous totalement incapable de travailler ou d'accomplir des tâches habituelles chez vous ?	1	2	3	4

Au cours de la semaine passée	Pas du tout	Un peu	Assez	Beaucoup
8. Avez-vous eu le souffle court ?	1	2	3	4
9. Avez-vous eu mal ?	1	2	3	4
10. Avez-vous eu besoin de repos ?	1	2	3	4
11. Avez-vous eu des difficultés pour dormir ?	1	2	3	4
12. Vous êtes-vous senti(e) faible ?	1	2	3	4
13. Avez-vous manqué d'appétit ?	1	2	3	4
14. Avez-vous eu des nausées (mal au cœur) ?	1	2	3	4
15. Avez-vous vomi ?	1	2	3	4
16. Avez-vous été constipée ?	1	2	3	4

**Au cours de la semaine passée**	**Pas du tout**	**Un peu**	**Assez**	**Beaucoup**
17. Avez-vous eu de la diarrhée ?	1	2	3	4
18. Etiez-vous fatigué(e) ?	1	2	3	4
19. Des douleurs ont-elles perturbées vos activités quotidiennes ?	1	2	3	4
20. Avez-vous eu des difficultés à vous concentrer sur certaines choses par exemple pour lire le journal ou regarder la télévision ?	1	2	3	4
21. Vous êtes-vous senti(e) tendu(e) ?	1	2	3	4
22. Vous êtes-vous fait du souci ?	1	2	3	4
23. Vous êtes-vous senti(e) irritable ?	1	2	3	4
24. Vous êtes-vous senti(e) déprimé(e) ?	1	2	3	4
25. Avez-vous eu des difficultés pour vous souvenir de certaines choses ?	1	2	3	4
26. Votre état physique ou votre traitement médical vous ont-ils gêné(e) dans votre vie FAMILIALE ?	1	2	3	4
27. Votre état physique ou votre traitement médical vous ont-ils gêné(e) dans votre vie SOCIALES (par exemple sortir avec des amis, aller au cinéma,…)	1	2	3	4
28. Votre état physique ou votre traitement médical vous ont-ils causé des problèmes financiers ?	1	2	3	4

Pour les questions suivantes, veuillez répondre en entourant le chiffre entre 1 et 7 qui s'applique le mieux à votre situation.

29. Comment évalueriez-vous l'ensemble de votre ETAT PHYSIQUE au courant de la semaine passée ?

Très mauvais Excellent

1 2 3 4 5 6 7

30. Comment évalueriez-vous l'ensemble de votre QUALITE DE VIE au cours de la semaine passée ?

Très mauvais Excellent

1 2 3 4 5 6 7

Université de Liège

Sujet n° :   Date d'évaluation : …./ …. / 20…..

MMSE (Folstein, Folstein, & McHugh., 1975)

Traduction : Derouesné et al, 1999

Orientation

	Score maximum	Score
En quelle année sommes-nous?	1	
En quelle saison sommes-nous?	1	
Quel mois sommes-nous?	1	
Quel jour de la semaine sommes-nous?	1	
Le quantième sommes-nous?	1	
Dans quel pays sommes-nous?	1	
Dans quelle province sommes-nous?	1	
Dans quel(le) ville / village sommes-nous?	1	
Quel est le nom de cet hôpital?	1	
A quel étage sommes-nous?	1	

Apprentissage

	Score maximum	Score
Je vais vous citer trois mots, lorsque j'aurai terminé, vous devrez les répéter. Lisez les mots à raison de 1 mot / seconde. Faites-les répéter et notez chaque mot correct.<br><br>CITRON … CLE … BALLON	3<br><br>Comptez et notez le nombre d'essais	

Concentration

	Score maximum	Score
Voudriez-vous soustraire 7 de 100?<br>Du résultat vous soustrayez chaque fois 7 jusqu'à ce que je vous dise "stop".<br>Comptez 1 point par soustraction exacte.<br>(93 86 79 72 65) … … … … …	5	
Voulez-vous épeler le mot « MONDE » à l'envers ?<br>E D N O M	5<br><br>Score le plus élevé :	

Mémoire

	Score maximum	Score
Quels étaient les trois mots que vous deviez retenir? (citron, clé, ballon)	3	

Le langage

	Score maximum	Score
Montrez une montre. Quel est le nom de cet objet?<br>Montrez un crayon. Quel est le nom de cet objet?	1<br><br>1	
Ecoutez bien et répétez après moi: "pas de mais, de si ni de et"	1	
Prenez ce papier de la main droite (prend papier : 1 point), pliez-le en deux (plie papier: 1 point) et posez-le sur vos genoux (dépose papier: 1 point)	3	
Montrez la feuille de papier sur laquelle est écrit: "Fermez les yeux"<br>Faites ce qui est écrit sur ce papier.	1	
Ecrivez une phrase.	1	
Pouvez-vous recopier cette figure?	1	

Score total (0-30) : …..

Fermez les yeux

ECRIVEZ UNE PHRASE

……………………………………

POUVEZ-VOUS RECOPIER CETTE FIGURE

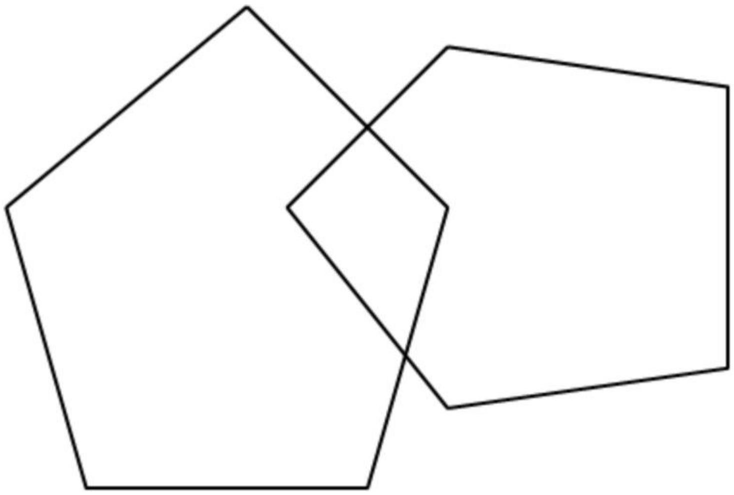

Univ de Université de Liège

Nom et prénom : Date évaluation: …. /…. /20 ….

A remplir sur base du dossier médical

Informations oncologiques
1. Date du diagnostic : __/__/____<br>2. Diagnostic : ………………<br>3. Carcinoma staging : ………<br>4. Si le patient a un cancer du poumon, est-il fumeur ? OUI – NON<br>5. Type de diagnostic :<br>○ Nouveau diagnostic<br>○ Progressive<br>○ Récidive

Charlson Index de Comorbidité

**Comorbidités**	**Présence**	**Points**
1. Infarctus du myocarde		1
2. Insuffisance cardiaque congestive		1
3. Maladie vasculaire périphérique		1
4. Accident vasculaire cérébral (sauf hémiplégie)		1
5. Démence		1
6. Maladie pulmonaire chronique		1
7. Connectivite		1
8. Ulcères oeso-gastro-duodénaux		1
9. Maladie hépatique légère		1
10. Diabète (sans complications)		1
11. Diabète avec atteinte d'organe cible		2

12. Hémiplegie		2
13. Maladie rénale modérée ou sévère		2
14. Autre tumeur solide (non métastatique)		2
15. Leucémie		2
16. Lymphome, myélome multiple		2
17. Maladie hépatique modérée ou sévère 3		3
18. Autre tumeur métastasée		6
19. SIDA		6
**SCORE TOTAL (0-37)**		

FOLLOW-UP

Traitement thérapeutique

	NON	OUI		SETTING
**Chirurgie**				○ Curative<br>○ Palliative<br>○ NR
**Chimiothérapie**			Laquelle :………………..<br>……………………………..<br>……………………………..	○ Curative<br>○ Néo-adjuvante<br>○ Adjuvante<br>○ Palliative<br>○ NR
**Radiothérapie**				○ Curative<br>○ Néo-adjuvante<br>○ Adjuvante<br>○ Palliative<br>○ NR
**Hormonothérapie**				○ Néo-adjuvante<br>○ Adjuvante<br>○ Palliative<br>○ NR
**Autre**			Laquelle :………………<br>……………………………..<br>……………………………..<br>……………………………..	○ Curative<br>○ Néo-adjuvante<br>○ Adjuvante<br>○ Palliative<br>○ NR
